# Distal renal tubular acidosis in lymphoplasmacytic lymphoma Waldenström’s macroglobulinemia: a case report

**DOI:** 10.4076/1757-1626-2-7198

**Published:** 2009-07-22

**Authors:** Harm HH Feringa, Afrooz Ardestani, Joseph Gnanaraj

**Affiliations:** Department of Medical Education, Internal and Preventive Medicine, Griffin Hospital and Yale University School of MedicineUSA

## Abstract

We present a case of distal renal tubular acidosis and acute renal insufficiency in a patient with Waldenström’s macroglobulinemia. Distal renal tubular acidosis has been described in hypergammaglobulinemia, but not in patients with Waldenström’s macroglobulinemia. To our knowledge, this is the first report to describe a possible association between distal renal tubular acidosis and Waldenström’s macroglobulinemia. Our case also emphasizes the importance of prompt recognition of distal renal tubular acidosis in patients with Waldenström’s macroglobulinemia because of its metabolic disturbances and potentially life threatening complications.

## Introduction

Waldenström’s macroglobulinemia is a lymphoproliferative disease characterized by the excessive production of IgM monoclonal protein. It is estimated that each year 1400 new cases occur in the United States [1]. Chronic renal insufficiency is unusual in Waldenström’s macroglobulinemia and is mostly caused by IgM deposits in the glomerular capillaries, interstitial infiltration of lymphoid cells, or amyloidosis [2-5]. In addition, distal renal tubular acidosis (RTA) has been described in hypergammaglobulinemia [6,7], but not in patients with Waldenström’s macroglobulinemia. We present a case of long-standing distal renal tubular acidosis (RTA) in a patient with Waldenström’s macroglobulinemia who came to our hospital because of acute renal dysfunction. In this case, Waldenström’s macroglobulinemia may have been a major contributor to her long-standing distal renal tubular acidosis and possibly to her acute renal dysfunction.

## Case presentation

An 84-year old Caucasian female was admitted to our hospital with muscle cramps and increasing lethargy during the past few days. Her medical history was significant for Waldenström’s macroglobulinemia, hypertension, paroxysmal atrial fibrillation, hyperlipidemia, and chronic kidney disease. Waldenström’s macroglobulinemia was previously diagnosed by serum-, urine- and immuno-electrophoresis revealing monoclonal production of IgM protein and flow cytometry demonstrating kappa restricted monoclonal B-cells with typical immunophenotype. A review of medical records also revealed that the patient had a long-standing history of distal hyperchloremic non-anion gap metabolic acidosis and high urinary pH, suggestive of distal RTA. She has then progressively developed nephrocalcinosis and renal dysfunction.

Prior to admission, the patient was not started on any new medications. On physical examination, the patient was alert and oriented with mild pedal edema on both legs. There was no musculoskeletal pain or rash. Examination of the cardiorespiratory system and abdomen was unremarkable. Admission laboratory results are demonstrated (Table 1). The patient presented with acute renal dysfunction and a mixed anion gap and non-anion gap metabolic acidosis. There was also an elevated total protein level with an elevated globulin fraction. Distal RTA was confirmed based on the non-anion gap metabolic acidosis and inappropriately high urinary pH.

**Table 1.** Laboratory results on hospital admission

Variable	Reference range	On admission
Glucose, mg/dL	70-105	87
Sodium, meq/L	136-145	147
Potassium, meq/L	3.5-5	4.6
Chloride, meq/L	98-106	120
Bicarbonate, meq/L	23-28	6
Anion gap	5-16	21
Blood urea nitrogen, mg/dL	8-20	61
Creatinine, mg/dL	0.7-1.3	2.4
Lactic acid, mmol/L	0.67-1.8	0.6
Calcium, mg/dL	9-10.5	9.5
Total protein, g/dL	6.0-7.8	10.9
Globulin, g/dL	2.5-3.5	7.3
Albumin, g/dL	3.5-5.5	3.6
Arterial blood gas		
- pH	7.38-7.44	7.10
- pCO2, mmHg	35-45	31
- pO2, mmHg	80-100	88
Urine					
- pH	5.0-8.0	6.0
- Protein, mg/dL	<30	100
- Osmolality, mosm/kg	300-1000	360
- Sodium, meq/L	-	37
- Potassium, meq/L	-	18.1
- Creatinine, mg/dL	-	79.4

The patient was given bicarbonate and her metabolic acidosis improved. Systemic lupus erythematosous, Sjögren’s syndrome, and rheumatoid arthritis were excluded by negative anti-double stranded DNA antibodies, antinuclear antibodies, and rheumatoid factor. Cryoglobulins were not detected. Serum viscosity was 2.0 centipoises (normal: ≤1.5 centipoises). The patient was negative for hepatitis B and C infection. Her liver, thyroid, and parathyroid function were intact. A computed tomography scan of the abdomen and pelvis demonstrated a right sided kidney stone with moderate right sided hydroureter. Serum and urine electrophoresis confirmed a persistent discrete monoclonal component of IgM kappa (40.7 g/L, normal: 0.2-1.4 g/L) in the gamma region.

During the hospital course, the patient developed pulmonary edema, pleural effusions, and basilar infiltrates suggestive of atelectasis. Left ventricular ejection fraction was >60% on echocardiography. Her oxygenation improved with intravenous diuretics and pulmonary nebulizer treatment. The patient remained lethargic during the first week of hospitalization and total parenteral nutrition was initiated. A regular diet was resumed after her mental status returned to baseline. Finally, her renal function improved and her estimated glomerular filtration rate returned to baseline. A renal biopsy was not obtained because of her improved renal function and increased bleeding risk with Coumadin therapy. After a hospital course of 3 weeks, the hyperchloremic metabolic acidosis resolved and she was successfully discharged to an extended care facility. Follow-up tests 2 weeks later, however, revealed a persistent hyperchloremic metabolic acidosis suggestive of distal renal tubular acidosis.

## Discussion

Distal (classic or type 1) RTA is a familiar or acquired disorder of the distal nephron characterized by failure to lower urinary pH resulting in a hyperchloremic metabolic acidosis. The underlying mechanism is not well understood but proposed mechanisms include a defective proton pump (secretory defect), an unfavorable electrical gradient for H+ secretion (voltage defect) and back diffusion of H+ or bicarbonate (gradient or permeability defect) [8]. Distal RTA can also lead to nephrolithiasis secondary to hypercalciuria, hyperphosphaturia, hypocitraturia, and low urine pH [9]. A urine pH >5.5 in the presence of non-anion gap systemic metabolic acidosis is diagnostic for distal RTA. Additional features supportive of the diagnosis include a positive urine anion gap and a urine sodium >25 meq/L [8]. In classic distal RTA, potassium levels are normal or decreased. However, hyperkalemic or voltage-dependent distal RTA also exists and can be explained by an inability to maintain a negative intraluminal voltage which promotes hydrogen and potassium secretion [8].

Our patient presented with severe lethargy and a mixed anion-gap and non-anion gap metabolic acidosis which were ascribed to uremia and distal RTA, respectively. The patient’s age and clinical presentation made an inherited form of distal RTA unlikely. Her past medical history of hyperchloremic metabolic acidosis was suggestive of chronic distal RTA. Screening for auto-immune disorders, the major causes of distal RTA, was negative. The patient had not been started on any new medications that could have explained her presentation. We therefore concluded that dysproteinemia secondary to Waldenström’s macroglobulinemia may be a major contributing factor to her long-standing chronic distal RTA and possibly to her acute renal dysfunction.

The most common clinical manifestations of Waldenström’s macroglobulinemia include weakness, fatigue, weight loss and chronic oozing of blood from the nose and gums [10,11]. Hyperviscosity-related symptoms include lethargy, which is often seen when serum viscosity exceeds 4 centipoises [12]. Renal disease is infrequently seen in Waldenström’s macroglobulinemia and mostly presents as mild proteinuria and microscopic hematuria [4]. Renal injury characteristically results from IgM kappa or lambda granular intracapillary fibrillary deposits [3]. These deposits start as subendothelial deposits and may lead to occlusion of the capillary lumen. Veltman and colleagues demonstrated that renal failure in a patient with Waldenström’s macroglobulinemia was caused by IgM deposits along the glomerular basement membrane and glomerular capillaries with mesangiocapillary glomerulonephritis [5]. Martello and colleagues described a case of renal disease caused by intramembranous IgM, IgG, and C3 deposits [13]. In a minority of patients, macroglobulins may precipitate in the cold (cryoglobulins) leading to proteinuria and renal disease [2]. Literature on acute renal dysfunction in Waldenström’s macroglobulinemia, as was the case in our patient, is rare. Berkel and colleagues reported a patient with acute renal failure as initial manifestation of a malignant lymphoproliferative disorder with monoclonal light chain immunoglobulin production [14]. Acute renal failure after contrast medium administration in a patient with macroglobulinemia was described by Matsumoto and colleagues [15].

To our knowledge, the association between distal RTA and IgM hyperglobulinemia, such as Waldenström’s macroglobulinemia, has not been reported in the literature. Previous studies have demonstrated a rare association between hypergammaglobulinemia and distal RTA [6,7]. The most common renal histopathological lesions in hyperglobulinemic distal RTA are interstitial lymphocytic infiltrates with tubular atrophy and fibrosis [16]. In myeloma patients, monoclonal IgG production and myeloma cast nephropathy are mainly responsible for causing distal tubulo-interstitial lesions and distal RTA [3]. Cast nephropathy in Waldenström’s macroglobulinemia is unlikely because of the low quantity of urinary monoclonal light chains. In Waldenström’s macroglobulinemia, interstitial infiltration with malignant lymphoid cells is common and present in around 60% of patients [4]. Unfortunately, no renal biopsy was obtained in our patient. It therefore remains unclear whether distal RTA in Waldenström’s macroglobulinemia was caused by IgM light-chain deposition or by lymphocytic infiltration.

## Conclusions

Our case illustrates that patients with Waldenström’s macroglobulinemia may present with distal RTA. Screening for distal RTA in these patients may be recommended, especially since the investigations are simple and can be done with blood and urine chemistry testing. Our case also suggests that Waldenström’s macroglobulinemia may be a major contributing factor to the development of distal RTA, and that these patients can develop acute renal dysfunction. Early recognition and timely treatment of these patients may prevent metabolic disturbances and potentially life threatening complications.

## Abbreviation

RTA: renal tubular acidosis
